# The connection between childhood maltreatment and eating disorder psychopathology: a network analysis study in people with bulimia nervosa and with binge eating disorder

**DOI:** 10.1007/s40519-021-01169-6

**Published:** 2021-03-28

**Authors:** Alessio Maria Monteleone, Orna Tzischinsky, Giammarco Cascino, Sigal Alon, Francesca Pellegrino, Valeria Ruzzi, Yael Latzer

**Affiliations:** 1 Department of Psychiatry, University of Campania “Luigi Vanvitelli”, Largo Madonna Delle Grazie, 80138 Naples, Italy; 2 Behavioral Science, Emek-Yezreel College, Emek-Yezreel, Israel; 3 Section of Neurosciences, Department of Medicine, Surgery and Dentistry ‘Scuola Medica Salernitana’, University of Salerno, Salerno, Italy; 4 Psychiatric Division, Rambam, Health Care Campus, Eating Disorders Institution, Haifa, Israel; 5 Faculty of Social Welfare and Health Sciences, University of Haifa, Haifa, Israel

**Keywords:** Childhood maltreatment, Emotional abuse, Bulimia nervosa, Binge eating disorder, Network analysis, Psychopathology

## Abstract

**Purpose:**

Childhood maltreatment (CM) experiences are associated with heightened risk of Eating disorders (EDs). The psychopathological pathways promoting this association in people with Bulimia nervosa (BN) and in those with Binge eating disorder (BED) are under-investigated.

**Methods:**

One hundred and eighty-one people with BN and 144 with BED filled in the Eating Disorder Inventory-2, to measure ED psychopathology, and the Childhood Trauma Questionnaire, to assess their early traumatic experiences. Network analysis was conducted to investigate the interplay between those variables. The shortest pathways function was employed to investigate the shortest out of all routes conveying the association between CM and ED-specific symptoms.

**Results:**

In both people with BN and with BED, all CM types were connected to the ED psychopathology through the emotional abuse node. The association between emotional abuse and ED-specific symptoms (bulimia and body dissatisfaction) differed in the two groups: in people with BN, it included ineffectiveness, while in people with BED, it involved impulsivity. Interoceptive awareness, an indirect measure of emotion regulation, was included in these pathways in both groups.

**Conclusion:**

In the light of literature showing that emotional abuse has a connecting role between CM and ED psychopathology also in anorexia nervosa, the present findings support the idea that emotional abuse conveys such association in all the main ED diagnoses. Ineffectiveness and impulsivity may represent the specific psychopathological dimensions connected to emotional abuse and promoting the maintenance of ED-specific symptoms in BN and in BED, respectively. These findings are worth of attention by clinicians.

**Level of evidence:**

Level III: evidence obtained from well-designed cohort or case–control analytic studies

**Supplementary Information:**

The online version contains supplementary material available at 10.1007/s40519-021-01169-6.

## Introduction

Eating disorders (EDs) are complex psychiatric illnesses likely originating by the interplay of psycho-social, biological, and genetic factors [1]. A recent umbrella review [2] assessing longitudinal data on pathogenetic risk factors showed that, among psycho-social factors, childhood sexual abuse and appearance-related teasing victimization are the factors more significantly associated with bulimia nervosa (BN) and any type of ED, respectively. Literature cross-sectional studies have confirmed that every type of childhood maltreatment (CM) experience is associated with EDs [3, 4] with a dose-dependent effect between CM load and symptoms severity [4]. In this line, recent studies corroborated the association between CM and EDs from multiple levels of analyses. In particular, decreased gray matter volumes and altered connectivity between brain regions were detected in maltreated people with EDs when compared to both no maltreated individuals and healthy peers [5]. The functioning of the hypothalamus–pituitary–adrenal axis was impaired in terms of cortisol awakening response [6] and cortisol response to an acute social stress [7] in acute or recovered [8] maltreated ED individuals. Experimental [7] and review [9] findings have suggested that CM may confer a higher vulnerability to develop ED symptoms in response to traumatic events in adulthood. This evidence contributed to the possibility of identifying a *maltreated ecophenotype* also in people with EDs as in other psychiatric disorders [10].

Despite this suggested importance of CM in EDs, the psychopathological pathways mediating the association between each CM type and ED-specific symptoms have been under-investigated. Emotion dysregulation [11, 12], reduced self-esteem [13] or dissociation [14] have been found as the possible mediating factors in people with anorexia nervosa (AN) or with BN. More recently, Rodgers et al. [15] indicated that depressed mood was an important driver for ED psychopathology in abused ED individuals.

Few studies have explored the association of CM and psychopathology in people with BED and showed either no association [16, 17] or significant association [18] with a possible mediating role of self-criticism [19, 20]. The use of different investigation methods and the assessment of different types of CM and of different outcomes (general or specific psychopathology) possibly contributed to the inconsistency of these findings. A novel method to explore the association between CM and ED psychopathology is the network analysis approach. This method differs from traditional statistics (i.e., multiple regression analysis) given that it identifies the connection between two variables, which points to the association between those variables taking into account the effects of all the other variables included in the network [21, 22]. Thus, network analysis simultaneously evaluates the association between each CM type and several ED psychopathology features taking into account the co-variance among ED symptoms and providing a more comprehensive evaluation of this association. Furthermore, the employment of a specific network function, the shortest pathways analysis, allows to identify the shortest route between external factors, which are conditions outside the psychopathological network but not outside the person (the “external field”, [21]), and the variables included in the network. We employed this method in a previous study [23] and found that all CM types were connected to ED psychopathology through emotional abuse, which in turn was connected to ED-specific symptoms through reduced interoceptive ability and ineffectiveness in people with restricting type AN and in patients with binge-purging symptoms, respectively. In the present study, we aimed to explore the association between each CM type and ED-specific symptoms through the use of the shortest pathways analysis in people with BN and in those with BED. On the basis of previous studies employing this method in people with AN and with binge-purging symptoms [23] and considering that emotional abuse was the CM type more frequently associated with psychopathology in people with BED [17–19, 24, 25], we hypothesized that emotional abuse should convey the association between each CM type and ED-specific symptoms in both study populations. In the light of literature findings [16, 19, 23, 25], our secondary hypothesis was that ineffectiveness lies in the shortest pathway between emotional abuse and ED-specific symptoms.

## Methods

### Participants

Consecutive patients attending the Eating Disorder Center of the Department of Psychiatry at the University of Campania “Luigi Vanvitelli” and of the Rambam Health care campus, Psychiatric division and Eating Disorder center, Haifa, Israel were recruited. Inclusion criteria for the study were: (1) age ≥ 18 years; (2) current diagnosis of BN or BED, according to DSM-5 and confirmed by the Structured Clinical Interview for DSM-5 Disorders-Research Version (SCID-RV) [26]; (3) absence of current/lifetime comorbid diagnosis of schizophrenia, bipolar disorder or substance abuse disorder; (4) absence of non ED-related severe physical disorders; (5) willingness to cooperate in the procedure and to sign a written informed consent.

Diagnostic assessment was made by a senior (in Israel) or trained (in Italy) psychiatrist (A.M.M.), who made the diagnosis first through a face-to-face clinical interview and then employing the SCID-RV, to confirm the ED diagnosis and psychiatric comorbidity.

### Procedure

Sociodemographic and clinical data were collected as part of the routine assessment of patients with EDs. Participants in the study were asked to complete the following questionnaires before entering specific treatment programs: (1) the Eating Disorders Inventory-2 (EDI-2) [27]; (2) the Childhood Trauma Questionnaire (CTQ) [28].

The EDI-2 [27] evaluates ED symptoms and psychopathology. The questionnaire includes 11 subscales: ineffectiveness, social insecurity, drive to thinness, interoceptive awareness, maturity fear, body dissatisfaction, perfectionism, interpersonal distrust, impulsivity, bulimia, and ascetism. Cronbach’s values ranged from 0.72 (maturity fear) to 0.92 (ineffectiveness).

The CTQ [28] investigates a self-report recall of childhood maltreatment experiences. It is a 28-item questionnaire which differentiates five types of CM: emotional neglect (EN) (“the failure of caretakers to meet children’s basic emotional and psychological needs, including love, belonging, nurturance, and support”; cut-off score ≥ 15; Cronbach’s *α* = 0.89), emotional abuse (EA) (“verbal assaults on a child’s sense of worth or well-being or any humiliating or demeaning behavior”; cut-off score ≥ 10; Cronbach’s *α* = 0.84*),* sexual abuse (SA) (“sexual contact or conduct between a child younger than 18 years and an adult or older person”; cut-off score ≥ 8; Cronbach’s *α* = 0.86), physical neglect (PN) (“the failure of caretakers to provide for a child’s basic physical needs, including food, shelter, clothing, safety, and health care”; cut-off score ≥ 8; Cronbach’s *α* = 0.72) and physical abuse (PA) (“bodily assaults on a child by an adult or older person that posed a risk of or resulted in injury”; cut-off score ≥ 8; Cronbach’s *α* = 0.87). The cut-off scores have been defined to indicate the occurrence of each CM type [30]. To the purposes of this study, a dimensional approach was adopted by employing the sum-score of each sub-scale [29]. Indeed, the use of continuous measures to evaluate trauma is largely documented in literature studies [9] and also employed in network analysis studies [31].

The study was approved by the Institutional Board of the University of Campania L. Vanvitelli and by the Ministry of Health and the Rambam Hospital’s Helsinki Ethics committee (40-09RAM).

### Statistical analysis

Differences between people with BN and those with BED were analyzed by an independent samples *t* test and the chi-squared test, where appropriate.

Network analysis (NA) was performed through R, version 3.4.4, using qgraph package [32]. A network is composed of nodes, which represent each variable included in the network, and edges, which are the connections among them. We have included in the network the EDI-2 and the CTQ sub-scores. The thickness of an edge graphically represents the magnitude of the association. We have estimated partial-correlation networks, where the association between two nodes is controlled for the influence of all other variables [32]. We have run two separate networks: one for the sample with BN and one for the BED group. To retain only meaningful associations, we applied a “least absolute shrinkage and selection operator” (LASSO) regularization [33], which shrinks small partial correlations and sets them to zero [34]. The Extended Bayesan Information Criterion (EBIC) [35], a parameter that sets the degree of regularization/penalty applied to sparse correlations, was set to 0.5 in these analyses. The accuracy of edge-weights was estimated by drawing bootstrapped confidence intervals calculated through “nonparametric” bootstrapping (nboots = 2500) [36]. The bootnet package [37] was used for this analysis.

Subsequently, we performed the shortest pathways analysis in each network. This kind of network detects the shortest path between two nodes, i.e., the quickest out of all the routes connecting these two nodes. The shortest path between two nodes represents the minimum number of steps needed to go from one node to the other [38], computed using Dijkstra’s algorithm [39]. We employed this method to identify the shortest paths between each CM node, as assessed by the CTQ, and the ED specific symptoms (body dissatisfaction and bulimia), evaluated by means of the EDI-2. The undirected edges assessed in this network point to conditional dependence between two variables: the edge-weight parameters reflect the strength of the associations between variables, which in turn suggests potential causal relationships [31, 36].

## Results

### Sample characteristics

Clinical characteristics of the study samples are reported in Table 1. One hundred and eighty-one people with BN and 144 people with BED were included in the study. One hundred and eleven (59%) people with BN have participated in a recent study [23] aiming to assess the shortest pathways between each CM type and ED-specific symptoms in patients with binge-purging (people with BN or with AN binge-purging type) or restricting behaviors (people with AN).

**Table 1:** Clinical characteristics of the diagnostic groups

	People with BN (*n* = 181)	People with BED (*n* = 144)	Mann–Whitney	*p*
Age	28.41 ± 9.31	37.33 ± 13.59	14,128.5	< 0.001
Age at onset	18.81 ± 5.78	23.23 ± 12.07	10,128.0	0.05
Illness duration, years	9.54 ± 8.31	13.15 ± 10.74	10,341.0	0.01
Body mass index	23.66 ± 6.18	35.86 ± 8.42	19,786.0	< 0.001
EDI-2				
Ineffectiveness	12.85 ± 7.89	9.58 ± 7.32	10,040.5	< 0.001
Maturity fear	8.75 ± 5.72	6.68 ± 5.02	10,458.5	< 0.001
Social insecurity	7.71 ± 4.30	5.24 ± 3.31	8697.5	< 0.001
Body dissatisfaction	16.02 ± 7.25	18.88 ± 6.13	16,325.0	< 0.001
Perfectionism	7.23 ± 4.60	6.03 ± 4.43	11,247.0	0.01
Interpersonal distrust	6.77 ± 4.81	4.97 ± 4.33	10,318.5	< 0.001
Impulsivity	9.86 ± 7.22	5.43 ± 5.17	8350.0	< 0.001
Drive for thinness	15.55 ± 5.27	12.81 ± 5.63	9225.5	< 0.001
Bulimia	10.58 ± 5.64	8.13 ± 5.18	9.971.0	< 0.001
Interoceptive awareness	14.02 ± 6.89	8.54 ± 6.12	7398.0	< 0.001
Asceticism	9.23 ± 4.46	6.61 ± 3.68	8777.0	< 0.001
CTQ				
Emotional neglect	13.30 ± 4.83	11.76 ± 4.90	10,838.0	< 0.01
Emotional abuse	10.21 ± 4.71	9.96 ± 4.47	12,970.0	0.71
Sexual abuse	6.69 ± 3.61	6.55 ± 3.34	12,973.5	0.85
Physical neglect	7.28 ± 2.51	6.97 ± 2.21	12,632.0	0.43
Physical abuse	6.89 ± 3.08	6.25 ± 2.51	11,718.0	< 0.05
Trauma, yes (%)			*χ*^2^	
Emotional neglect	76 (42)	43 (29)	5.31	0.02
Emotional abuse	83 (45)	66 (45)	0.01	0.94
Sexual abuse	47 (25)	28 (19)	2.49	0.11
Physical neglect	68 (37)	49 (34)	0.51	0.47
Physical abuse	38 (21)	27 (19)	0.10	0.75

A current comorbid anxiety disorder was detected in 73 subjects (43 with BN and 30 with BED), while 101 individuals (51 with BN and 50 with BED) were diagnosed with a comorbid depressive disorder. The Mann–Whitney test (Table 1) showed that, compared to people with BED, people with BN reported younger age and lower BMI values and scored higher in all EDI-2 sub-scores, except for body dissatisfaction, which was higher in people with BED. CTQ emotional neglect and physical abuse scores were higher in BN people than in BED individuals. The chi-squared test confirmed a higher prevalence of emotional neglect events in people with BN than in those with BED.

### Network analyses

**Fig. 1:**
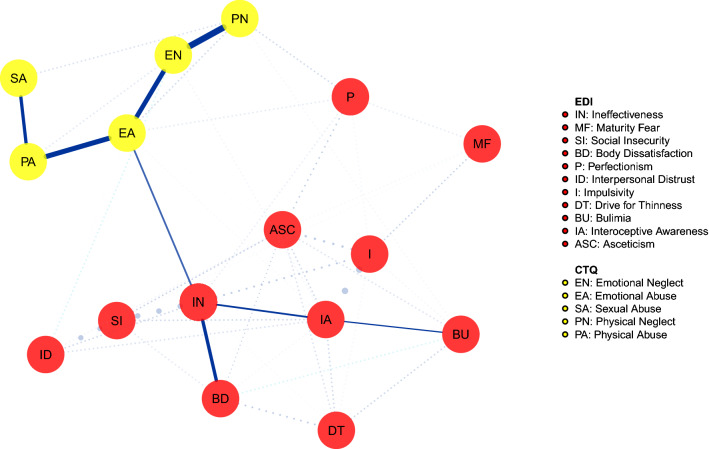
Shortest pathways network in the bulimia nervosa group. Network illustrating the shortest path between childhood maltreatment dimensions and eating disorders symptoms in the bulimia nervosa group. Continuous lines = shortest pathway of interest. Dotted lines = edges not included in the shortest pathways. For color version, see this figure online

The shortest pathways analysis identified the shortest route between the CTQ nodes and the EDI-2-specific (bulimia and body dissatisfaction) symptoms. The network in Fig. 1 shows the connections between each childhood trauma experience and the EDI-2 sub-scores in the group with BN. It emerged that there were no direct connections between CM nodes and ED symptoms, except for the connection between emotional abuse and ineffectiveness. This node was directly connected with the ED-specific symptom body dissatisfaction, while the shortest path to reach bulimia included the interoceptive awareness.

**Fig. 2:**
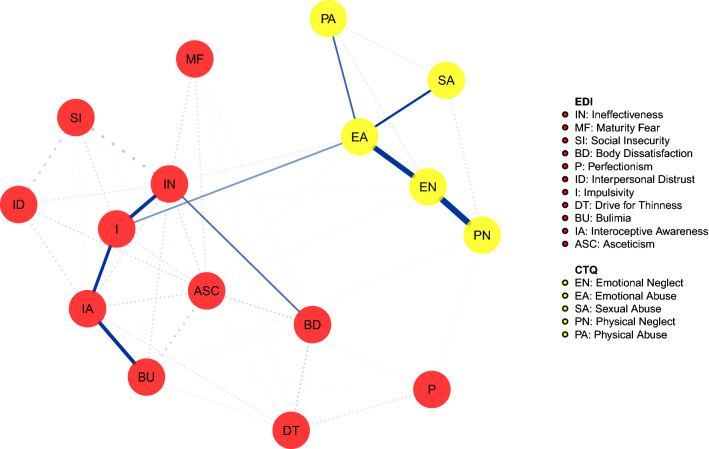
Shortest pathways network in the binge eating disorder group. Network illustrating the shortest path between childhood maltreatment dimensions and eating disorders symptoms in the binge eating disorder group. Continuous lines = shortest pathway of interest. Dotted lines = edges not included in the shortest pathways. For color version, see this figure online

The network in Fig. 2 illustrates the connections between childhood trauma experiences and EDI-2 in the group with BED. As in the previous network, CM nodes were not connected with ED symptoms, except for emotional abuse that was connected with impulsivity. This symptom, in turn, was positively associated with body dissatisfaction via ineffectiveness, while the shortest route to bulimia included the interoceptive awareness.

The bootstrapped confidence intervals of estimated edge-weights are reported in Supplementary Figures 1 and 2.

## Discussion

According to our first study hypothesis, the emotional abuse experience is included in the shortest route between all CM types and ED psychopathology in both people with BN and those with BED. These populations, instead, differed in relation to the second study hypothesis. Indeed, in the BN group ineffectiveness and interoceptive awareness were included in shortest pathway between emotional abuse and specific symptoms; in the BED group, also impulsivity was included in that pathway and was directly connected to emotional abuse.

The employment of the shortest pathways analysis allowed us to highlight the central role of emotional abuse in the association between CM and ED psychopathology. This kind of early abuse refers to experiences of being humiliated or receiving self-demeaning behaviors [28]. Bullying or appearance-related teasing experiences as well as physical or sexual abuse events may promote emotional abuse. These findings confirm the importance to evaluate childhood emotional abuse when assessing CM in people with BN or with BED, despite sexual abuse has been longitudinally recognized as the unique risk factor for EDs [2] and despite literature highlighting the association of physical and sexual abuse with EDs [3]. In this line, emotional abuse was found to be the only CM type directly associated to ED psychopathology, without any effect of psychiatry comorbidity [40] and previous studies suggested that different types of CM do not have the same impact on ED symptoms [41, 42]. Furthermore, in people with AN and in those with BN, the emotional trauma was the specific CM type contributing to the heightened emotional stress reactivity and to the increased food-related and body concerns in response to a social stress test [7]. In people with BED, several studies have found that emotional abuse was higher than in obese people without BED [18] and was the unique CM type associated to psychopathology [17–19, 25, 43]. However, none of these studies assessed the association between emotional abuse and psychopathology taking into account the effects of the other CM types and the covariation among symptoms, as done in the present network analysis. Only one previous study [23] has employed the same method in patients with restricting type AN and in patients with binge-purging symptoms and has revealed that emotional abuse was the node conveying the effects of each CM experience on ED psychopathology. Taken together, these results point to emotional abuse as the CM type promoting the association between CM and psychopathology across all the principal ED diagnoses.

The second main finding of this study is that ineffectiveness and interoceptive awareness are included in the shortest pathways between emotional abuse and ED-specific symptoms in people with BN, while also impulsivity was involved in these pathways in individuals with BED. In people with BN, ineffectiveness was directly associated with body dissatisfaction and was associated to bulimic symptoms through the interoceptive awareness node. According to the EDI-2 questionnaire [27], we found that inadequacy, loneliness, and empty feelings may be associated with lower accuracy to detect and regulate inner and emotional states, which in turn are associated with bulimic symptoms. Although causality cannot be inferred, the observed pathway is consistent with models describing ED behaviors as maladaptive strategies to cope with emotions [44, 45]. Furthermore, these findings are in line with literature data highlighting the role of ineffectiveness as mediating the association between CM and ED psychopathology in BN [13].

Similarly to people with BN, in those with BED ineffectiveness was connected to body dissatisfaction. However, in the BED group, impulsivity instead of ineffectiveness was connected to emotional abuse. Thus, the connection between emotional abuse and impulsivity may represent a specific pathway promoting psychopathology in people with BED, while the same role was played by ineffectiveness in BN and by interoceptive awareness in people with AN restricting type [23]. Impulsivity is a multidimensional personality trait spanning a cognitive component named reward sensitivity, a behavioral dimension related to inhibition and an affective component [46, 47]. The BED has been conceptualized as a phenotype within the obese spectrum characterized by heightened impulsivity [48–50] with increased rash-spontaneous behavior in general and, specifically, toward food [51]. Furthermore, impulsivity is associated with negative urgency [52], that is the need to regulate negative emotions engaging in rash actions and risky behaviors [53], especially in ED patients with food addiction [54]. This suggests that bulimic ingestion of food can be conceived as an attempt to escape unbearable emotions [54, 55]. This theory may be supported by our network pathway showing a connection between impulsivity, lower interoceptive awareness and bulimic symptoms. Furthermore, the association of this pattern with emotional abuse is in line with studies showing that impulsivity mediates the association between childhood abuse and disordered eating in a non-clinical population [56].

Limitations of this study need to be acknowledged. First, the cross-sectional nature of these data does not make possible to infer causality regarding the connections identified in the networks and does not allow to distinguish their state or trait nature. Second, the CTQ provides a self-report assessment of childhood adverse experiences that is possibly affected by recalling bias, without characterization of the specific nature of the trauma or the period when it occurred. Third, the lack of inclusion in the network of general psychiatric symptoms, which may be advisable in the light of high rate of psychiatry comorbidity in people with BN and with BED [1, 48], precludes a complete assessment of the connections between CM and the whole psychopathology of BN and BED. Finally, no stability measures have been provided to assess the reliability of the identified shortest pathways, given that a stability test has not been yet developed.

The main strength of this study is that it allows to explore, for the first time in people with BN and with BED, the associations between different CM types and ED symptoms taking into account for the effects of each CM type and for covariation between symptoms and showing the shortest out of all routes between CM and ED symptoms.

## Conclusions

Emotional abuse represents the CM experience lying in the pathway between early adverse events and ED psychopathology in people with AN [23], with BN or with BED. Thus, emotional abuse may be conceptualized as a possible common experience through which all CM types converge on ED psychopathology in all principal ED groups. This should prompt clinicians as well as researchers to investigate the childhood emotional abuse experiences in people with EDs. However, specific ED psychopathological variables seem to connect emotional abuse and ED symptoms in each ED group: interoceptive awareness in people with AN [23]; ineffectiveness in those with BN and impulsivity in those with BED. The network theory [17] suggests that the interplay between nodes may maintain a state of prolonged activations of further symptoms in the network. Therefore, these specific pathways may be taken into account in treatment and prevention programs. Reduced self-esteem and high impulsivity are central factors in the psychopathology of BN and of BED [48, 57]: these findings suggest clinicians to evaluate their connections with early traumatic experiences and with trauma related maladaptive schemas and to address these connections in psychotherapy interventions.

## What is already known on this subject?

All kinds of childhood maltreatment are associated with an increased risk to develop an eating disorder. People with eating disorders and history of childhood maltreatment show a more severe form of the illness. The pathways promoting the association between childhood maltreatment and eating disorder psychopathology have been under-investigated.

## What does this study add?

In people with bulimia nervosa and with binge eating disorder, all types of childhood maltreatment are associated to psychopathology through the emotional abuse experience. The shortest pathways conveying the association between emotional abuse and eating disorder specific symptoms include ineffectiveness in bulimia nervosa and impulsivity in binge eating disorder.

## Supplementary Information

Below is the link to the electronic supplementary material.

Supplementary file1 (PDF 27 KB) Bootstrapped confidence intervals of edge-weights in the bulimia nervosa group.

Supplementary file2 (PDF 26 KB) Bootstrapped confidence intervals of edge-weights in the binge eating disorder group.

## Data Availability

The data that support the findings of this study are available from the corresponding author upon reasonable request.
