# Supplementary figures and images for: The connection between childhood maltreatment and eating disorder psychopathology: a network analysis study in people with bulimia nervosa and with binge eating disorder
Source: Eat Weight Disord. 2021 Mar 28;27(1):253–61. doi: 10.1007/s40519-021-01169-6 (PMC8860810; doi:10.1007/s40519-021-01169-6)

● Bootstrap mean    ● Sample

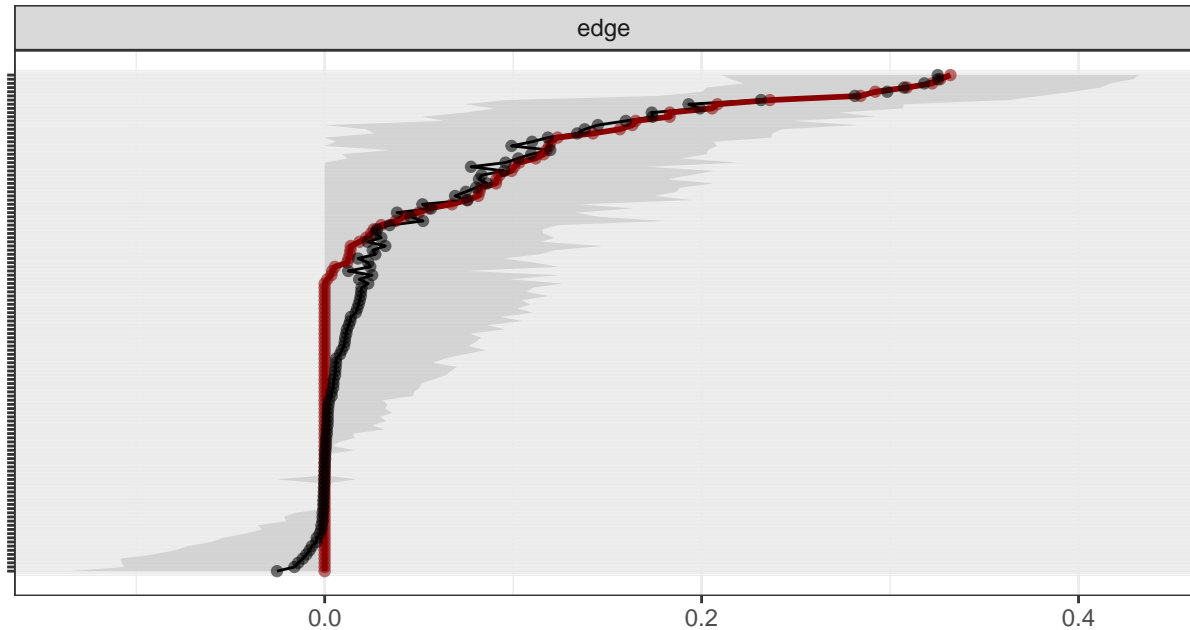

Supplement: Supplementary file 1 — Supplementary file1 (PDF 27 KB) Bootstrapped confidence intervals of edge-weights in the bulimia nervosa group. [file 40519_2021_1169_MOESM1_ESM.pdf]

● Bootstrap mean    ● Sample

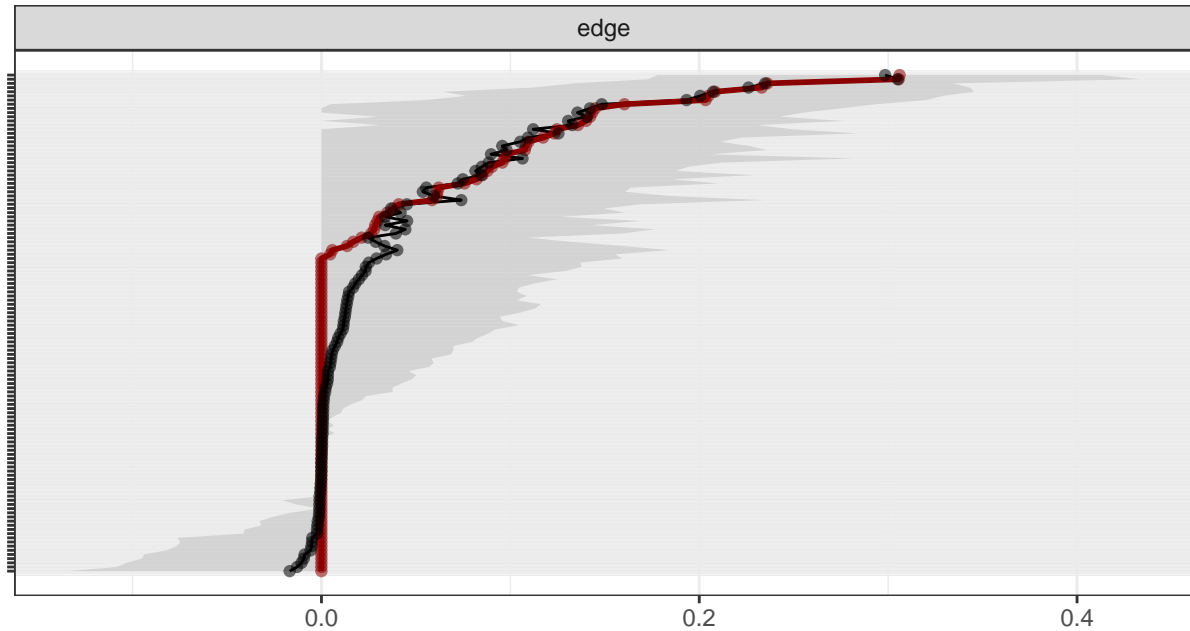

Supplement: Supplementary file 2 — Supplementary file2 (PDF 26 KB) Bootstrapped confidence intervals of edge-weights in the binge eating disorder group. [file 40519_2021_1169_MOESM2_ESM.pdf]
